# Advanced Computer Vision-Based Subsea Gas Leaks Monitoring: A Comparison of Two Approaches

**DOI:** 10.3390/s23052566

**Published:** 2023-02-25

**Authors:** Hongwei Zhu, Weikang Xie, Junjie Li, Jihao Shi, Mingfu Fu, Xiaoyuan Qian, He Zhang, Kaikai Wang, Guoming Chen

**Affiliations:** 1 Centre for Offshore Engineering and Safety Technology, China University of Petroleum, Qingdao 266580, China; 2 Department of Building Environment and Energy Engineering, The Hong Kong Polytechnic University, Kowloon, Hong Kong, China; 3 PipeChina West Pipeline Company, Urumqi 830000, China; 4 School of Emergency Management and Safety Engineering, China University of Mining and Technology (Beijing), Beijing 100083, China

**Keywords:** subsea gas leak monitoring, optical camera detection, advanced computer vision, faster R-CNN, YOLOv4

## Abstract

Recent years have witnessed the increasing risk of subsea gas leaks with the development of offshore gas exploration, which poses a potential threat to human life, corporate assets, and the environment. The optical imaging-based monitoring approach has become widespread in the field of monitoring underwater gas leakage, but the shortcomings of huge labor costs and severe false alarms exist due to related operators’ operation and judgment. This study aimed to develop an advanced computer vision-based monitoring approach to achieve automatic and real-time monitoring of underwater gas leaks. A comparison analysis between the Faster Region Convolutional Neural Network (Faster R-CNN) and You Only Look Once version 4 (YOLOv4) was conducted. The results demonstrated that the Faster R-CNN model, developed with an image size of 1280 × 720 and no noise, was optimal for the automatic and real-time monitoring of underwater gas leakage. This optimal model could accurately classify small and large-shape leakage gas plumes from real-world datasets, and locate the area of these underwater gas plumes.

## 1. Introduction

As offshore oil and gas prospecting and exploiting move into deep-water fields and sensitive areas, issues such as subsea equipment failures, seabed pipeline leakages, submarine gas eruptions, etc., result in the increasing number of subsea natural gas leak events [1]. Once a subsea gas leak occurs, the forming gas plume will bring the flammable natural gas and oil into the atmosphere, disperse them around the offshore platforms or working vessels, and ultimately pose a potential fire and explosion environment. So far, numerous fire and explosion accidents with heavy casualties have occurred; these have resulted in economic losses and environmental pollution, such as the Deepwater Horizon explosion accident in 2010 [2], the Elgin Platform gas leak accident in 2012 [3], and the Pemex platform fire accident in 2021 [4]. In addition to the possibility of fire and explosion accidents, the releasing gas plume is likely to disorder the maneuverability and stability of nearby vessels due to strong surface currents [5,6]. Furthermore, the release of oil and gas serves to produce a kind of hydrocarbon mixture gas, primarily methane, which aggravates the marine environment and ecosystem [7,8]. On the other hand, a large amount of CO_2_ gas has been injected and stored in the appropriate geological reservoirs of the subsea. Due to injection facility failure and seal failure, there exists a certain risk of CO_2_ gas leaks in deep subsea that would inevitably destroy the marine ecosystem [9,10,11,12]. Consequently, subsea oil and gas leakage is gradually a safety problem for the offshore oil and gas industry and creates an environmental problem for the global climate and marine ecosystem.

To address the above problems, the critical working regions, such as offshore platforms and vessels, should be monitored for subsea gas leaks so that related operators can raise the corresponding alarm at an early stage and thereby make timely emergency and prevention measures. Past decades have witnessed the development of subsea gas leak monitoring approaches and technologies [13,14,15,16,17,18,19,20,21,22,23]. These works can be classified into two categories: software-based internal methods and hardware-based external methods [14,22]. Among them, the internal method mainly monitors related gas flow parameters, such as flow mass or volume, negative pressure waves, and gas flow rate, and then judges whether a subsea gas leak is occurring [14,19,22]. By contrast, the external method needs external sensors such as hydrophones [13,19], optoelectronic sensors [17,18], fiber-optic cables [15,16,23], and optical cameras [14,20,21,24] to realize the subsea gas leak monitoring. Compared to the external method, the internal methods have some drawbacks [14,19]. Firstly, such monitoring methods can only be used for the leakage detection of subsea pipelines. Secondly, they cannot be applied for the leakage detection of small-size gas plumes. Thirdly, they make it difficult to pinpoint the location of the leakage source. Thereby, most research has been on the improvement and innovation of external methods.

Of these external methods, the optical camera-based method can provide an intuitive monitoring video for subsea scenarios as leaked gas plumes can be detected through the operator’s visual inspection and the corresponding leak source can be pinpointed. Although this method is limited to subsea light conditions and water turbidity, its monitoring effect can be advanced by equipping it with an additional light source [14,20] or applying other methods, such as the laminar flow approach [25,26] and the infrared output camera [27]. In this way, this method is always applied for subsea monitoring tasks such as spilled oil and gas tracking using the autonomous buoy system (SOTAB-I) [28,29], the submarine visual information system [30,31], and the released gas plume and bubble imaging system [32,33]. In this monitoring application, the operator’s visual inspection and judgment of monitoring video frames is still a critical prerequisite for finding and warning of abnormal subsea events. Such human intervention would not only result in high labor costs, it would also create a high false alarm rate for the monitoring effect [34,35]. To address these problems, some literature on anomaly detection based on monitoring videos performs the computer vision approach to realize an automated detection for anomaly events without human intervention, while achieving excellent detection efficiency and detection accuracy. Likewise, as in subsea gas leak monitoring, an automated intelligence approach will greatly improve monitoring accuracy and efficiency.

In terms of automated anomaly detection, advanced computer vision-based approaches have become a promising alternative to learning about the visual features of varied abnormal events and how to intelligently detect these events. Previous works have pointed out that the convolutional neural network (CNN) is capable of effectively extracting the spatial features of image datasets and accurately learning these features. Therefore, for industrial anomaly detection, researchers have proposed many automatous and intelligent methods for real-time anomaly detection, such as natural gas leak detection [35,36], machine fault detection [37,38], and structure crack detection [39,40]. These works realize the automatic detection of anomaly events without manual intervention, which greatly improves the effectiveness and accuracy of anomaly detection. Unfortunately, classic CNN architectures are only suitable for anomaly classification; that is, they can tell if an anomaly event occurs, but they cannot give the position or range of the anomaly event, which is not conducive to the emergency handling of abnormal events. To realize both classification and localization for anomaly detection, in recent years, several advanced computer vision-based detection approaches have been advanced, such as the Faster Region Convolutional Neural Network (Faster R-CNN) [39], Single Shot Multi-Box Detector (SSD) [40], and the YOLO series [41]. Of these, the Faster R-CNN and the YOLO series both have a relatively high detection speed and accuracy, and so have been widely applied in varied industrial anomaly detection tasks, such as oil and gas leak detection [42,43], surface defects detection [44,45] and machine fault detection [46,47]. However, to the authors’ well-known knowledge, very limited research has been conducted on the application of such an advanced computer vision-based approach for automated subsea gas leak monitoring. Furthermore, which is the best out of the Faster R-CNN and the YOLO series is still unknown.

This paper aimed to propose an automatous and intelligent monitoring method integrating the advanced computer vision approach with a subsea optical camera for gas leak detection. Meanwhile, a relatively systemic comparison analysis was conducted to determine which of the Faster R-CNN and the YOLO series is more suitable for actual subsea gas leak scenarios. Firstly, an underwater gas leak experiment was conducted for achieving a large number of video datasets characterizing the gas plume feature, which was used to develop monitoring models. Secondly, two open available videos about subsea oil and gas leaks were selected as real-world subsea gas leak scenarios to assess and compare the application performance of these models developed from the experimental dataset. Furthermore, a sensitivity analysis of varied image sizes and noise intensity datasets on method performance was also conducted. A comparison of detection accuracy and speed for the two models developed from the different datasets was performed. This study provided a methodology and technology guidance for constructing a subsea oil and gas device with a long-term automatic and real-time monitoring system.

## 2. Theory of Two Advanced Computer Vision-Based Detection Approaches

### 2.1. Faster R-CNN Approach

**Figure 1:**
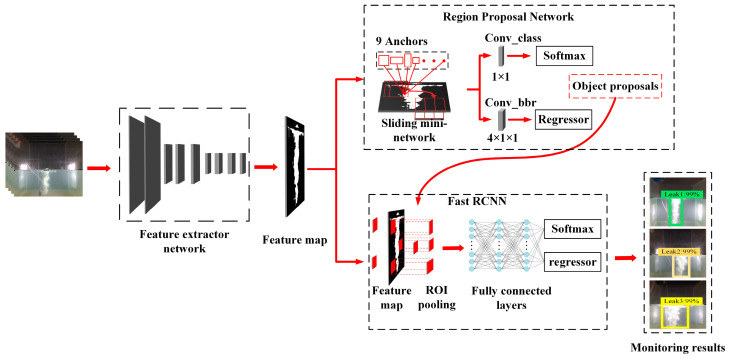
The detection architecture of the Faster R-CNN approaches.

The Faster R-CNN [39] is one of the most popular two-stage object detection approaches, which can classify and pinpoint objects in one scene with an almost real-time detection speed and a comparatively high detection accuracy. From Figure 1, this Faster R-CNN approach is composed of a feature extractor network, a region proposal network (RPN), and a Fast R-CNN module. Of these, the feature extractor network is responsible for extracting feature maps representing object information from the inputting image data, then the RPN roughly generates region proposals that contain objects from these extracted feature maps; eventually, the Fast R-CNN module precisely classifies object proposals and refines object spatial locations from feature information integrating the generated region proposal and extracted feature map. Due to the two-stage detection structure design composed by the RPN and the Fast R-CNN module, the Faster R-CNN shows a strong object feature extracting and learning ability, and so has a relatively high accuracy detection result among most object detection approaches. Meanwhile, to improve detection speed, this approach introduces a new region proposals generation module RPN instead of the sliding window algorithm in the previous generation R-CNN approaches. Because the RPN is essentially a small fully connected network that can keep an extremely fast production speed for the region proposal, the Faster R-CNN approach can realize an almost real-time detection speed.

The development of a detection model based on the Faster R-CNN approach is to determine and fine-tune weights in all neural network layers by alternate training between the RPN module and the Fast R-CNN module. The RPN module is first trained under feature maps extracted by a pre-trained feature extractor network for producing the region proposal containing the object. Then, the Fast R-CNN module is trained by object region proposals generated by the trained RPN module to accurately detect the object classification and location. Lastly, fixing the shared convolutional layers and fine-tuning the unique convolutional layers creates two modules that share the same convolutional layers and form a unified network. The RPN and Fast R-CNN training are both based on region proposals or object classification and location. As such, the training loss function of the RPN and the Fast R-CNN is a multi-task loss function integrating the classification loss and regression loss. Equations (1) and (2) describe the loss function of the RPN module and the Fast R-CNN module, respectively.

(1) $$L(\{p_{i}\},\{t_{i}\})=\frac{1}{N_{cls}}\sum_{i}L_{cls}(p_{i},p_{i}^{*})+\lambda \frac{1}{N_{reg}}\sum_{i}p_{i}^{*}L_{reg}(t_{i},t_{i}^{*})$$

(2) $$L(p,u,t^{u},v)=L_{cls}(p,u)+\lambda \left[u\geq 1\right]L_{reg}(t^{u},v)$$

where i is the index of an anchor in a mini-batch and $p_{i}$ is the predicted probability of anchor i being an object. If the anchor i is positive, the ground-truth probability $p_{i}^{*}$ is 1. Instead, the ground-truth probability $p_{i}^{*}$ is 0. The $t_{i}$ is a vector including the four coordinates of the predicted bounding box, and $t_{i}^{*}$ is the ground-truth box coordinate vector of the positive anchor. $L_{cls}$ is the classification loss defined by log term, while $L_{reg}$ is the bounding box regression loss defined by smooth $L_{1}$. x,y,w, and h represent the center coordinates, width, and height of the predicted bounding box or adjusted anchor, respectively. $N_{cls}$ and $N_{reg}$ denote the mini-batch size and the anchor locations number, which are used to balance the classification loss and regression loss with the parameter λ [31].

### 2.2. YOLOv4 Approach

**Figure 2:**
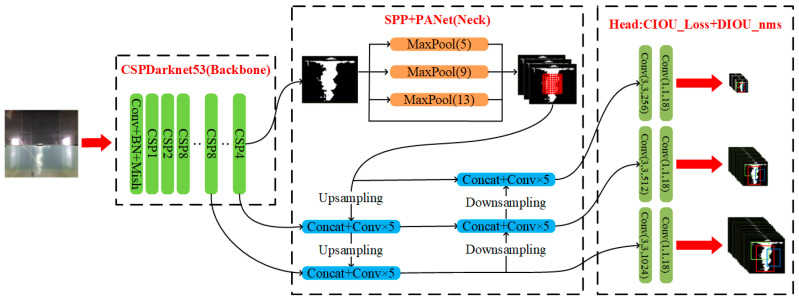
The overall YOLOv4 architecture.

The YOLOv4 [41] approach faces an actual monitoring application that requires a fast detection speed and relatively high accuracy. Compared to the Faster R-CNN, YOLOv4 applies one-stage detection architecture composed of backbone, neck, and head layers, which is shown in Figure 2. Unlike the Faster R-CNN, it first generates region proposals containing the object through the RPN and then further precisely regresses. As well as classifying the object location and label by the Fast R-CNN, the YOLOv4 directly extracts the object feature and conducts the object location regression and label classification. Specifically, the backbone layer first extracts the image feature map from a batch of outputting images to learn some object features. Secondly, to improve object detection accuracy and avoid the disappearance of low-level features, the middle neck layer generally integrates multiple scales features (especially the low-level feature) and conducts many pooling operations to boost the receptive field of the feature map, which greatly enriches the transferring image feature to be prone towards learning about many image details. Finally, the last head layer accepts the enhanced feature information and conducts the bounding box regression, label classification, and confidence prediction.

To improve detection accuracy and efficiency, YOLOv4 focuses on integrating and innovating some advanced tricks, which can significantly improve detection accuracy and almost had less impact on the detection speed. Among these, the backbone layer applies the new feature extractor CSPDarknet53 according to the Mish activation function [48] and Cross Stage Partial Network (CSPNet) [49], which can boost the feature extraction ability of the network. In the neck layer, the spatial pyramid pooling (SPP) [50] structure is introduced to capture local information of the extracted feature map by four times max pooling and then incorporate this information, which benefits the enhancement of the receptive field and so improves detection performance, especially for small targets. Meanwhile, this layer also applies a path aggregation network (PANet) [51] to fuse multiple scale feature information, which can increase the semantic feature and the location feature. Besides, to overcome incomplete expression between the bounding box and ground truth, the detection head layer performs *CIOU* loss [52], replacing *IOU* loss as the location regression loss function, which is shown in Equation (3):

(3) $$CIOU\_Loss=1-IOU+\frac{\rho^{2}(b,b^{gt})}{c^{2}}+\alpha v$$

where b and $b^{gt}$ denote the central points of the predicted bounding box and ground truth bounding box, $\rho \left(\cdot \right)$ refers to the Euclidean distance, and c is the diagonal length of the smallest enclosing box covering the two boxes. α is the positive trade-off parameter, and v measures the consistency of the aspect ratio, whose mathematical formulas are shown in Equations (4) and (5).

(4) $$\alpha =\frac{v}{(1-IOU)+v^{\prime }}$$

(5) $$v=\frac{4}{\pi^{2}}{\left(\arctan\frac{w^{gt}}{h^{gt}}-\arctan\frac{w}{h}\right)}^{2}$$

## 3. Methodology to Develop an Optimal Gas Leakage Monitoring Model

**Figure 3:**
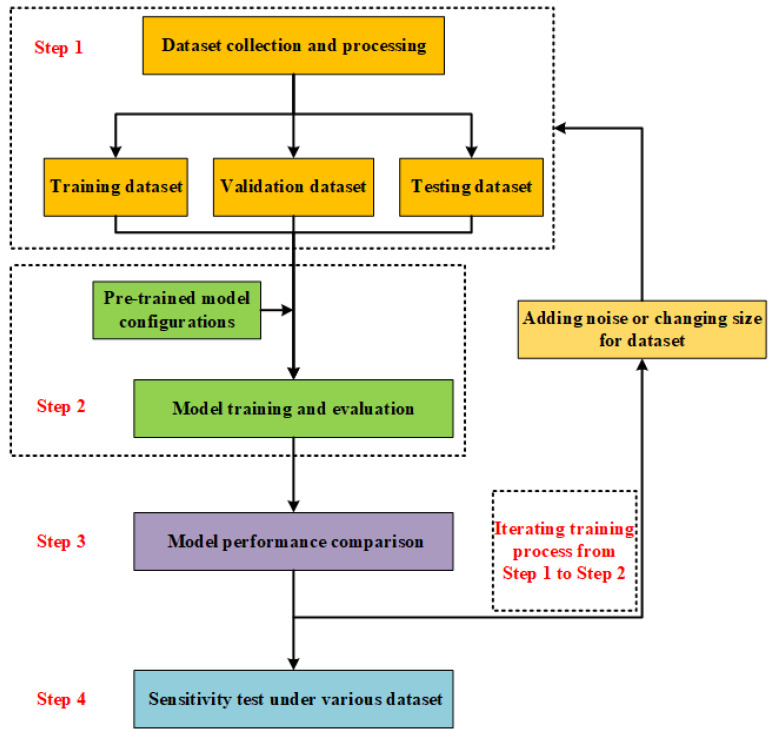
Developing flowchart regarding the optimal model for monitoring underwater gas leaks based on computer vision-based approaches.

Both the Faster R-CNN approach and YOLOv4 can perform real-time and online monitoring of underwater gas leaks, but they have their own advantages for monitoring accuracy and speed, respectively. The Faster R-CNN, a two-stage detector, is more accurate in terms of detection accuracy, but its detection speed is relatively slow. YOLOv4, a one-stage detector, has a faster detection speed due to its simple architecture, while its detection accuracy is relatively poor. In order to tradeoff accuracy and speed for monitoring underwater gas leakage, we need to develop an optimal gas leakage monitoring model from the two computer vision-based approaches. Figure 3 displays the developing flowchart regarding the optimal model for monitoring underwater gas leaks from the above-mentioned computer vision-based approaches. The detailed developing process is shown in Figure 3.

Step 1: We collected a large number of imaging datasets containing underwater gas leak features and then processed these datasets to construct the training dataset, the validation dataset, and the testing dataset for developing the optimal model for underwater gas leaks. The collection of imaging datasets was from underwater gas leak experiments or open-access videos. Furthermore, processing these imaging datasets using open-source software, namely LabelImg, generated the annotation datasets, including classifications of whether or not there was gas leakage and the location positions of the gas leakage plume. Finally, the imaging datasets and annotation datasets were integrated as the developing datasets (i.e., training dataset, validation dataset, and testing dataset) for developing the optimal monitoring model.

Step 2: Next, we developed the Faster R-CNN model and the YOLOv4 model on the basis of their own pre-trained model according to transfer learning. Transfer learning has been proven as an accurate and efficient approach for developing computer vision-based monitoring models by fine-tuning corresponding pre-trained models into training datasets. In order to save the optimal fine-tuned model, a validation step was used to evaluate whether the model’s performance improved after every fine-tuning. Through validation, the fine-tuned model could be saved when its performance was upgraded, which ensured achieving the optimal monitoring model after developing monitoring models. To date, relevant researchers and institutions have launched all kinds of pre-trained models. Hence, we selected a competitive pre-trained model for both the Faster R-CNN and YOLOv4 approaches, respectively, so as to develop a comprehensive monitoring model for underwater gas leakage.

Step 3: At the same time, we further explored the effect of noise intensity and image size of the developing datasets on the monitoring model’s accuracy and speed, to determine a comprehensive developing dataset for the optimal monitoring model. Assessing the model’s accuracy included assessing its classification accuracy and location accuracy. Hence, we calculated the mAP value to evaluate monitoring performance by opening tool MSCOCO API [53]. The MSCOCO API also provided the inference time. In addition, to evaluate the monitoring performance under real-world datasets, we calculated true positive (TP), false positive (FP), true negative (TN), and false negative (FN), to construct the ROC-AUC according to the literature [54].

Step 4: We further conducted a sensitivity analysis for exploring the effect of noise intensity and image size on model monitoring performance, to determine the optimal developing dataset. Furthermore, we evaluated and compared the monitoring performance of the Faster R-CNN and YOLOv4 models developed by the optimal datasets. Finally, we determined the optimal model for monitoring underwater gas leakage.

## 4. Collection Datasets Concerning Underwater Gas Leakage Plume

**Figure 4:**
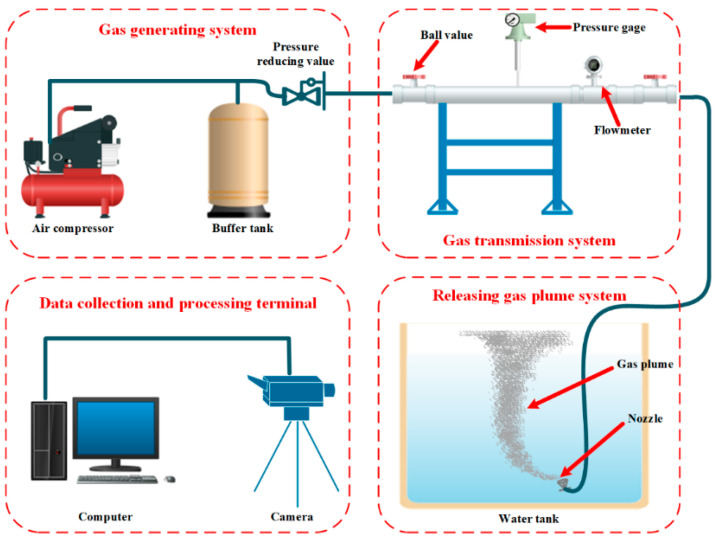
The overall architecture of the experiment system for underwater gas leakage.

Due to the shortage of gas leak datasets from real subsea accidents, an underwater gas leak experiment was conducted to collect a large number of datasets that included underwater gas leak features. The overall architecture of the experiment system is shown in Figure 4. This experiment system mainly included four modules: a gas generating system, a gas transmission pipeline, a gas leak water tank, and a data collection and processing terminal. Through this experiment system, the generating gas was transported into a tank and formed gas plumes in the water, and the data collection terminal recorded these gas plumes.

Table 1 presents the details of the experimental configuration. From this, we employed airflow to replace natural gas for experimental safety. We also set the air leakage pressures to 0.2, 0.4, and 0.6 MPa to produce small, medium, and large sizes gas plumes, respectively. Furthermore, during the experiments, the forming gas plumes were real-time recorded by a video camera with a video resolution of 1280 × 720 and a video spread of 25 frames/s; the shooting time of each video sequence was 120–180 s. Finally, we achieved a large number of video sequences recording the gas leakage plumes.

**Figure 5:**
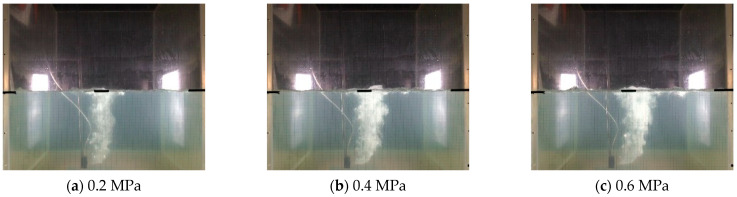
Images of underwater gas plumes under three leakage pressures.

**Figure 6:**
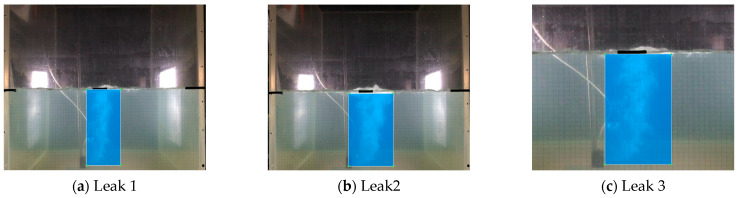
Annotation labels and ground truth boxes for underwater gas plumes under three leakage pressures.

Next, we segmented the recorded videos into images that included the underwater gas plumes, and in total, achieved 8622 images, including 2000 images under 0.2 MPa, 3000 images under 0.4 MPa, and 3622 images under 0.6 MPa. Figure 5 displays the underwater gas leakage plumes under three leak pressures. As for these images, we further annotated the gas leakage plumes to produce annotation datasets that included classification labels and ground truth box positions. Figure 6 displays the annotation labels and ground truth boxes of the gas plume images under three leakage pressures. As can be seen, gas plumes of 0.2 MPa were labeled Leak1, gas plumes of 0.4 MPa were labeled Leak2, and gas plumes of 0.4 MPa were labeled Leak3. Meanwhile, the ground truth boxes covered the gas plumes, which were used as location information. Accordingly, both the experimental gas plume images and the annotation datasets were integrated as the developing datasets for the Faster R-CNN and YOLOv4 models.

**Figure 7:**
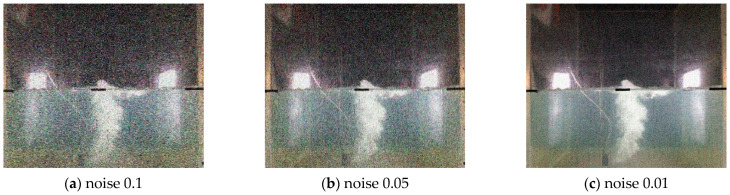
Images of underwater gas plumes under Gaussian noise with intensities 0.01, 0.05, and 0.1.

**Figure 8:**
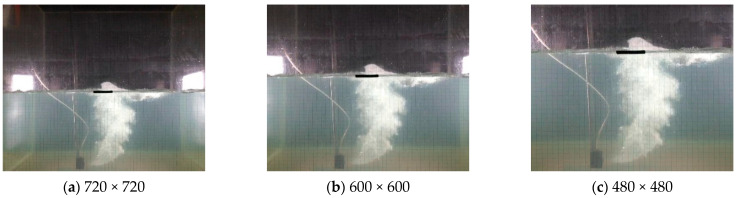
Images of underwater gas plumes under image sizes of (720 × 720), (600 × 600), and (480 × 480).

Furthermore, we processed the above images by adding Gaussian noises with intensities 0.01, 0.05, and 0.1 for achieving the developing datasets, respectively, and clipped image sizes of (720 × 720), (600 × 600), and (480 × 480) for achieving the developing datasets, respectively. Figure 7 displays images of the underwater gas plumes under three noise intensities, while Figure 8 displays images of the underwater gas plumes under three image sizes.

**Figure 9:**
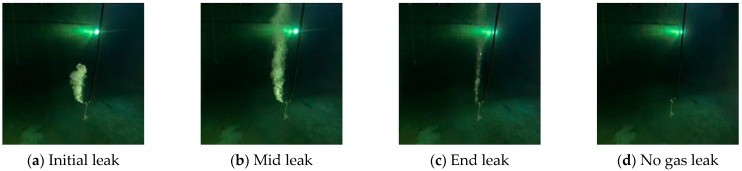
Images from Co. L. Mar. underwater gas leak experiments under different leakage stages.

**Figure 10:**
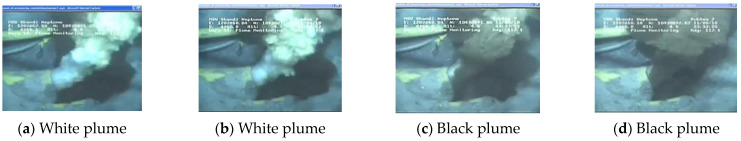
Images of white gas plumes and black oil plumes from BP subsea oil and gas leak accidents.

Apart from the experimental developing datasets, we also searched for open-access videos of underwater gas plume images from Co. L. Mar. experiments, and subsea oil and gas release images from BP leakage accidents. Figure 9 displays the underwater gas plumes under different leakage stages from the Co. L. Mar. experiments. Figure 10 displays images of oil and gas leakage plumes from the BP leakage accidents. From this, it can be seen that white-releasing gas plumes and black-releasing oil plumes exist.

In summary, in total, we collected nine developing datasets: an experimental developing dataset, three developing datasets adding Gaussian noises, three developing datasets with clipped image sizes, and two opening leakage datasets; see Table 2. As for the experimental developing datasets, noise developing datasets, and clipped developing datasets, we randomly selected 80%, 10%, and 10% of the datasets as the training datasets, validation datasets, and testing datasets, respectively, which were used for developing the monitoring model. The Co. L. Mar. and BP opening leakage datasets were used to evaluate the models’ monitoring performance for real leakage scenarios.

## 5. Results and Discussion

In order to develop an optimal model, we selected Faster_rcnn_inception_coco_v2 and YOLOv4.CONV.137 as the pre-trained model for the Faster R-CNN approach and the YOLOv4 approach according to related literature. Table 3 lists the detailed pre-trained model configurations. This model development process was carried out by a high-performance computer server with a configuration of 64 GB RAM, an i9-9900K CPU, and a NVIDIA GeForce RTX 2080Ti GPU card. By comparing the model performance under different developing datasets, we explored the effect of image size and noise intensity on the developing models’ performance. Then, we conducted a monitoring task for the Co. L. Mar. dataset and the BP accidental dataset to determine the optimal monitoring model.

### 5.1. Monitoring Performance Comparison of Two Approaches under Experimental Datasets

**Figure 11:**
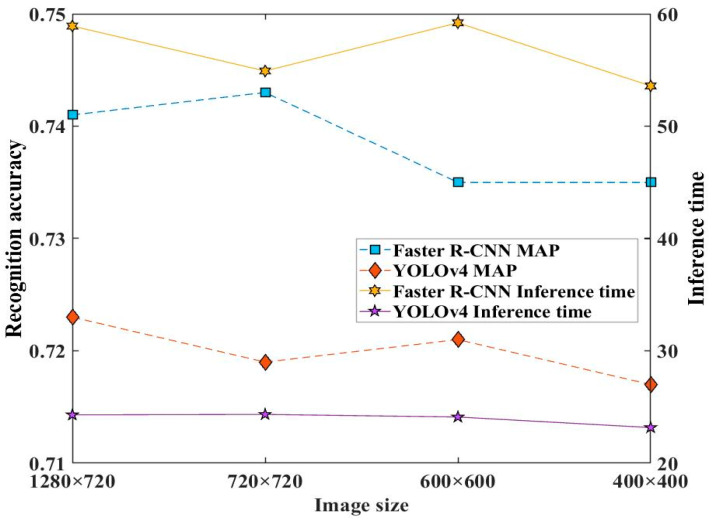
Effect of image size of developing datasets on the recognition accuracy and inference time of the two approach models.

Figure 11 displays the monitoring performance of the two approaches based on models developed under different image sizes experimental datasets. The recognition accuracy mAP value was calculated by MS COCO API. As can be seen, decreasing image size roughly resulted a reduction of the recognition accuracy mAP value. For example, the mAP value under image sizes 1280 × 720 and 720 × 720 was relatively higher than those of 600 × 600 and 480 × 480 for the Faster R-CNN model, and the mAP value of the YOLOv4 model under image size 1280 × 720 was maximum. Additionally, the inference time of the Faster R-CNN model fluctuated at 56 ms with the changing of image sizes, and that of the YOLOv4 model was about 23 ms, which was almost free from the image sizes. Accordingly, the large image size benefits the recognition accuracy of both the Faster R-CNN model and the YOLOv4 model.

**Figure 12:**
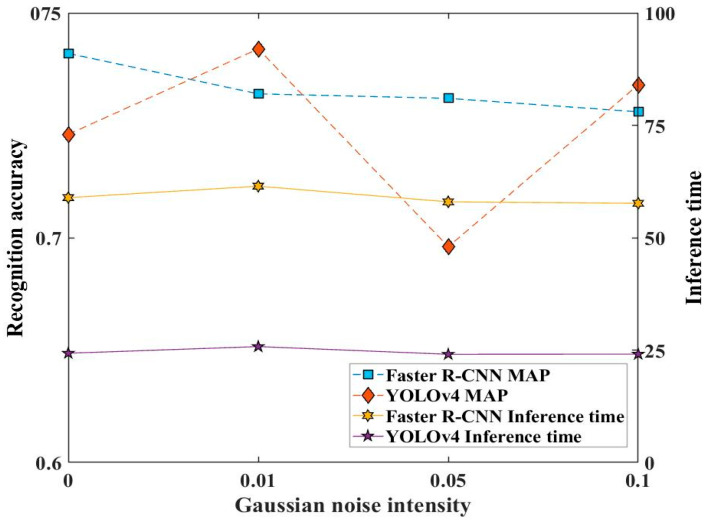
Effect of Gaussian noise of developing datasets on the recognition accuracy and inference time of the two approach models.

Figure 12 displays the monitoring performance of the two approaches under different noise intensities. It can be seen that the mAP value of the Faster R-CNN model declines with the increasing noise intensity, and that of YOLOv4 declines when 0.01 is at a maximum 0.742 and fluctuates at mAP value 0.742. Meanwhile, the inference times of the Faster R-CNN and YOLOv4 models were almost unaffected by noise intensities.

Additionally, by comparing the recognition accuracy and inference time of the two approaches, we found that the Faster R-CNN approach was better overall than the YOLOv4 approach in recognition accuracy and lower than the YOLOv4 approach in inference time. Accordingly, the image sizes 1280 × 720 were optimal for the two models under experimental datasets, a noise intensity of 0 was optimal for the Faster R-CNN model, and a noise intensity of 0.01 was optimal for the YOLOv4 model. In addition, both models could reach a real-time monitoring speed, which essentially was not affected by the image size and noise intensity in terms of inference time.

### 5.2. Monitoring Performance Comparison of Two Approaches under Real-World Datasets

**Figure 13:**
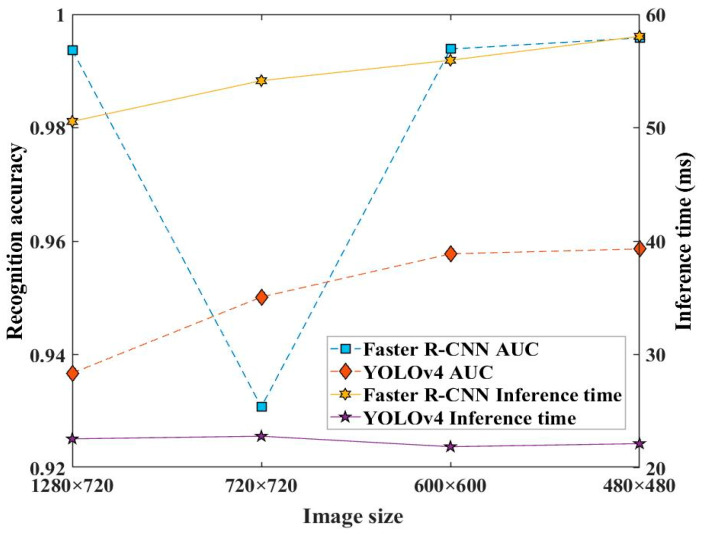
Effect of image size of developing datasets on the recognition accuracy and inference time of the two approach models.

Figure 13 displays the recognition accuracy and inference time for Faster R-CNN model and the YOLOv4 model under image sizes for the Co. L. Mar. underwater gas leakage datasets. The recognition accuracy represented the model’s classification accuracy calculated by ROC-AUC. It can be seen that the AUC value for image size 720 × 720 was 0.93076 for the Faster R-CNN model, while that for image size 1280 × 720 was 0.99368. Meanwhile, the AUC value for image size 480 × 480 was at its maximum of 0.95863. Additionally, from this figure, it can be seen that as the image size decreased, the inference time of the Faster R-CNN model had a large increase. Accordingly, the image size 1280 × 720 was suitable for developing the Faster R-CNN model while the image size 480 × 480 was suitable for the YOLOv4 model.

**Figure 14:**
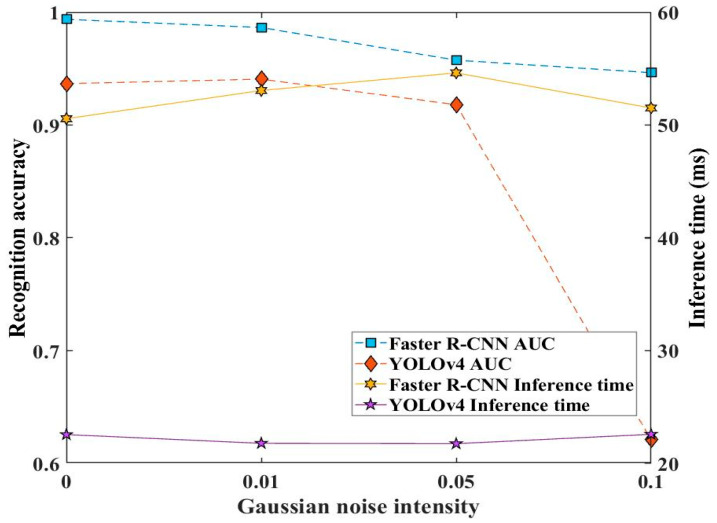
Effect of noise intensity of developing datasets on the recognition accuracy and inference time of the two approach models.

Figure 14 shows the recognition accuracy and inference time of the Faster R-CNN and YOLOv4 models developed by different noise intensity datasets for the Co. L. Mar. underwater gas leakage datasets. From this, it can be seen that as the noise intensity increased, the AUC value of the two approach models gradually decreased, and specifically, the AUC value of YOLOv4 rapidly dropped to 0.6212 at noise intensity 0.1. Meanwhile, the inference time of the two approach models was almost unaffected by noise intensity.

Accordingly, the image size 1280 × 720 was optimal for the Faster R-CNN model, the image size 480 × 480 was optimal for the YOLOv4 model, and the noise intensity 0 was optimal for the two models. In addition, both models could reach a real-time monitoring speed.

### 5.3. Comparison between Faster R-CNN Model and YOLOv4 Model under Real World Datasets

**Figure 15:**
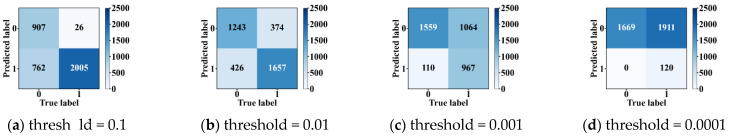
Confusion matrixes of the YOLOv4 models developed by the experimental dataset with image size 480 × 480 and noise intensity 0 under different pre-determined thresholds. Note that label 0 represents the existing gas leakage and label 1 represents no gas leakage. The predefined thresholds were determined according to the classification probability outputted by the YOLOv4 model.

**Figure 16:**
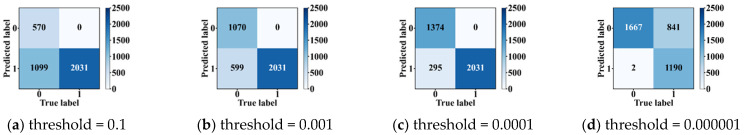
Confusion matrixes of the Faster R-CNN model by the experimental dataset with image size 1280 × 720 and noise intensity 0 under different pre-determined thresholds.

**Figure 17:**
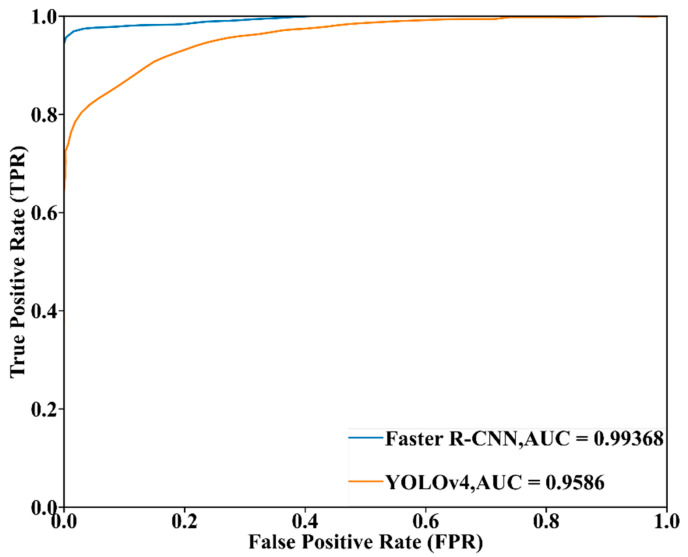
Comparison regarding ROC curve and AUC value between the Faster R-CNN model developed by the experimental dataset with image size 1280 × 720 and noise intensity 0, and the YOLOv4 model developed by the experimental dataset with image size 480 × 480 and noise intensity 0.

**Figure 18:**
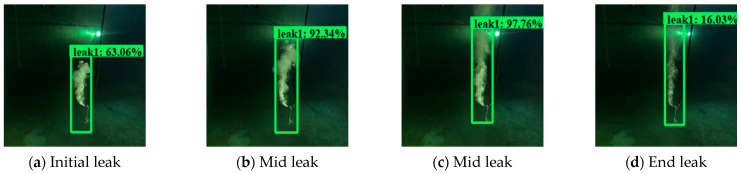
Visualization examples of the Faster R-CNN model developed by the image size 1280 × 720 dataset under the Co. L. Mar. dataset.

**Figure 19:**
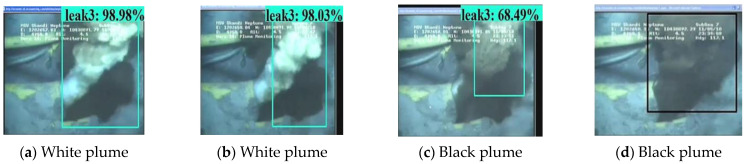
Visualization examples of the Faster R-CNN model developed under the image size 1280 × 720 dataset under the BP dataset.

**Figure 20:**
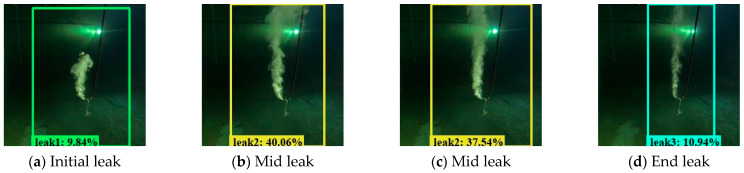
Visualization examples of the YOLOv4 model developed by the image size 480 × 480 dataset under the Co. L. Mar. dataset.

**Figure 21:**
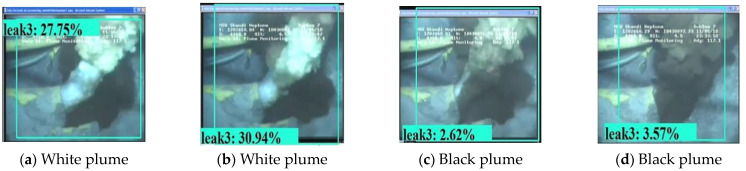
Visualization examples of the YOLOv4 model developed by the image size 480 × 480 dataset under the BP dataset.

Considering model performance under real-world datasets, the Faster R-CNN model under image size 1280 × 720 and noise intensity 0, and the YOLOv4 model under image size 480 × 480 and noise intensity 0, was optimal. Furthermore, we conducted a comparison of monitoring performance between the two models under the Co. L. Mar. and BP datasets, as shown in Figure 15, Figure 16, Figure 17, Figure 18, Figure 19, Figure 20 and Figure 21.

Figure 15 and Figure 16 display the comparison of confusion matrixes between the Faster R-CNN model and the YOLOv4 model. From these, it is clear that for the Faster R-CNN and YOLOv4 models, the number of FNs gradually decreases and the number of FPs gradually increases along with the decrease of the predefined thresholds; that is, the decreasing of the thresholds makes the monitoring model more prone to false alarm for normal scenarios. By comparing these, it is clear that the Faster R-CNN model can reach a trade-off between FP (0) and FN (295) when the threshold 0.0001, which indicates that for the Faster R-CNN model, the false alarm for the normal scenario never existed and some no alarm for the gas leakage existed. However, the YOLOv4 model performed a severe false alarm phenomenon under the low threshold and a severe no alarm phenomenon under the high threshold. Accordingly, the Faster R-CNN model was more accurate for monitoring gas leakage compared to the YOLOv4 model.

Figure 17 displays the comparison for the ROC curve and AUC value between the Faster R-CNN model developed by the experimental dataset with image size 480 × 480 and noise intensity 0, and the YOLOv4 model developed by the experimental dataset with image size 1280 × 720 and noise intensity 0. It can be seen that the AUC value of the Faster R-CNN model was 0.99368, and that of the developed YOLOv4 model was 0.95863, which indicates that the Faster R-CNN approach was more suitable for monitoring underwater gas leakage compared to the YOLOv4 approach. Meanwhile, the Faster R-CNN model’s ROC curve was above that of the YOLOv4 model, and the Faster R-CNN model’s ROC curve was closer to the Y axis than that of the YOLOv4 model. Such circumstances indicate that the Faster R-CNN model can keep a relatively high TPR under low FPR; that is, the Faster R-CNN approach can perform at high classification accuracy for gas leakage and keep has relatively little false alarm for normal scenarios. In this regard, the YOLOv4 model was worse than the Faster R-CNN model.

Figure 18 and Figure 19 display the visualization example of the Faster R-CNN model developed under image size 1280 × 720, while Figure 20 and Figure 21 display that of the YOLOv4 model developed under image size 480 × 480. As can be seen, the Faster R-CNN model was better than the YOLOv4 model in terms of classification and location for underwater gas plumes. On the one hand, the Faster R-CNN model classified the underwater gas plumes of the Co. L. Mar. datasets into Leak1 due to the small shape of the plumes, while the YOLOv4 model classified these plumes into Leak2. Meanwhile, the Faster R-CNN model provided a relatively higher classification probability than the YOLOv4 model, which indicated that the Faster R-CNN model was more confident for the small shape plumes of the Co. L. Mar. datasets. On the other hand, by observing the location box, it was found that although the Faster R-CNN model could not recognize the black plume seen in Figure 19, it could output a bounding box close to the area of the gas plume from real-world datasets, while the YOLOv4 model located no related areas in the monitoring datasets. This means that the Faster R-CNN model has better learning ability for features such as plume shape and color. Additionally, in terms of inference time, the Faster R-CNN model could keep a real-time monitoring speed of 56 ms/frame. Accordingly, the Faster R-CNN model developed by image size 1280 × 720 and no noise was optimal for monitoring underwater gas leakage.

Accordingly, the Faster R-CNN model is relatively better than the YOLOv4 model in terms of classification accuracy and visual location accuracy. As for the real-world datasets, the Faster R-CNN model can provide a larger classification likelihood for gas plumes and predict a more accurate location box for gas plumes. Hence, the developed monitoring model can be accurately applied for monitoring the gas leakage of real-world datasets. However, the predicted results of the monitoring model were uncertain predictions that failed to achieve robustness in the real-world scenario. To address this, probabilistic methods, especially Variational Bayesian Inference, need to be applied to the deep learning-based object detection model to model a probability distribution for prediction. With these, the uncertainty information for real-world scenarios can be quantified and it can be determined whether the prediction is credible.

## 6. Conclusions

In summary, this study proposed an advanced computer vision based underwater gas leakage approach. The comparison regarding monitoring performance of the advanced computer vision approaches for experimental and real world underwater gas leakage is conducted. The conclusions are as follows:

(1) Faster R-CNN model developed by experimental developing datasets with image size 1280 × 720 and no noise is optimal to real time and automatic monitor for underwater gas leakage from real world datasets.

(2) Compared to YOLOv4 approach, Faster R-CNN based optimal model performs a better learning ability for underwater gas plume features, and so the classification and location for gas plume is extremely accurate, especially distinguishing the small and large size of underwater leakage gas plume.

(3) Faster R-CNN based optimal model performs a better scenario adaptability for real world datasets. This means that Faster R-CNN based model could accurately locate the area of underwater gas plume, while YOLOv4 based model could roughly locate many unrelated areas of monitoring datasets.

However, the additional points of the proposed approach are required to be discussed.

(1) This computer vision based approach need a large number of manual annotation datasets, which causes in a huge time and human cost so as to limit the developing efficiency of monitoring model.

(2) This computer vision based approach perform a poor monitoring performance for some gas plume with black color, which can be solved by enriching the plume color in the developing datasets.

Overall, future works are expected to solve the above limitations and the publicly available developing dataset concerning subsea oil and gas leakage is welcome for the future works.

## Figures and Tables

**Table 1:** Experimental configuration for the underwater gas leak.

Experimental Medium	Leaking Pressure	Video Resolution	Video fps	Shooting Time
Airflow	0.2, 0.4, 0.6 MPa	1280 × 720	25 frames/s	120–180 s

**Table 2:** The corresponding datasets for developing and testing of monitoring model.

Dataset	Annotation Files	Image Size	Noise	Source
Original datasets	xml files	1280 × 720	none	Experiment
Noise_0.1	xml files	1280 × 720	0.1	Experiment
Noise_0.05	xml files	1280 × 720	0.05	Experiment
Noise_0.01	xml files	1280 × 720	0.01	Experiment
720 × 720	xml files	720 × 720	none	Experiment
600 × 600	xml files	600 × 600	none	Experiment
480 × 480	xml files	480 × 480	none	Experiment
Co. L. Mar. dataset	N/A	320 × 240	N/A	Real-world
BP dataset	N/A	480 × 360	N/A	Real-world

**Table 3:** Configuration of pre-trained models for the Faster R-CNN and YOLOv4 approaches.

Configuration	Faster R-CNN Approach	YOLOv4 Approach
Pre-trained model	Faster_rcnn_inception_coco_v2	YOLOv4.CONV.137
Feature extractor	Inception V2	CSPDarknet53
Dataset structure	COCO	VOC2018
Initial learning rate	0.0002	0.001
Batch size	1	64
Detection classes	3	3

## Data Availability

Not applicable.
